# Relationship of Momentary Volition to Occupational Experience and Life Perspective in Undergraduate Students

**DOI:** 10.3390/healthcare11182471

**Published:** 2023-09-05

**Authors:** Mi Young Hong, Sun-Wook Lee, Eun Young Kim

**Affiliations:** 1 Department of Occupational Therapy, Far East University, Eumseong-gun 27601, Republic of Korea; 4804848@hanmail.net; 2 Department of Occupational Therapy, Daegu University, Gyeongsan-si 38453, Republic of Korea; sun.w.lee@daegu.ac.kr; 3 Department of Occupational Therapy, Soonchunhyang University, Asan-si 31538, Republic of Korea

**Keywords:** the Model of Human Occupation, experience sampling method, occupational questionnaire, life balance, life satisfaction

## Abstract

Our lives are comprised of moment-to-moment activities and experiences. According to the Model of Human Occupation, our occupational experiences can be affected by volition, which consists of personal causation, values, and interests. This study investigated how momentary volition could affect activity satisfaction and mind-wandering while performing occupations. This study also examined the relationship of momentary volition with overall life perspectives on life satisfaction and life balance. Undergraduate students participated in this cross-sectional study. The experience sampling method (ESM), which repeatedly collects real-time data in everyday life, was applied in this study to measure students’ momentary states such as activity, volition, activity satisfaction, and mind-wandering. After completing the ESM, participants’ life satisfaction and life balance were measured using the Satisfaction With Life Scale (SWLS) and the Life Balance Inventory (LBI), respectively. Forty-two participants and 1092 sampling data points were included in the analysis. At the event level, regression analysis was performed to identify volition elements to contribute to activity satisfaction and mind-wandering. At the personal level, correlation analysis was used to determine the relationship of momentary volition to life satisfaction and life balance. Momentary personal causation, values, and interests contributed to activity satisfaction. Mind-wandering was predicted negatively by interests but positively by personal causation. Momentary interests were positively correlated with SWLS and LBI scores. This study demonstrated that momentary volition was associated with activity satisfaction and engagement, as well as life satisfaction and balance in undergraduate students. Momentary volition, especially interests, contributed to positive occupational experiences and life perspectives. This study suggests that occupational therapy practitioners need to consider momentary interests to provide occupation-centered interventions for undergraduate students.

## 1. Introduction

“Life takes on meaning in the minute-by-minute reality in which we experience ourselves achieving the ordinary things…”

—Gary Kielhofner (1949–2010) [1]

Human lives consist of occupations subjectively experienced by an individual. The Model of Human Occupation [2,3] is the conceptual practice model in occupational therapy, which explains how anticipating, choosing, experiencing, and interpreting one’s activities compose a volitional process in one’s given environment. A volitional process is a complex, ongoing, and patterned process involving one’s personal causation, values, and interests. Personal causation is the awareness of personal effectiveness, which includes a sense of personal capacity and self-efficacy. Values are personal convictions and a sense of obligation about what is meaningful to do, which is derived from culture. Interests are related to the pleasure of doing something and are reflected in a preference for certain activities over others. The moment-to-moment volitional process shapes the occupational narrative, which “ties together past, present, and future as well as integrate multiple themes of self and the world” [3]. To determine one’s life trajectory, understanding subjective experience and its associated perception can be a relevant factor. The Model of Human Occupation suggests that “minute-by-minute reality” becomes “life,” stating that “each person has unique occupational narrative or story of how a client’s volition, habituation, performance capacity, and environment interact over time to influence what a client does with his or her life” [3].

Most previous studies of subjective volition based on the Model of Human Occupation surveyed participants’ past typical days using the Occupational Questionnaire [4,5,6]. This retrospective report can portray a general occupational pattern and associated volitional components of participants’ activities. Previous studies have measured volition as a comprehensive construct [7,8,9]. Moment-to-moment activity experience has been emphasized in the theoretical model [1]. To our knowledge, no study has attempted to observe immediate volition in real-time contexts or examine the relative effect of each volitional component on occupational participation. Thus, this study addressed this research gap by measuring moment-to-moment volition and analyzing its relative contribution to occupation.

To understand volition while performing life activities, the present study applied an experience sampling method (ESM) [10]. This ESM can measure humans’ experiences in their actual life context by asking them to answer a series of questions at that moment. Since people report their current experiences, the ESM can provide ecologically valid information. The ESM was developed to study flow, which is the state of intense concentration on activities due to intrinsic motivation [11,12,13]. According to flow theory, a person with high skills and challenges can experience flow, which means that the person fully engages in the activity and enjoys it [14]. It has been thought that flow corresponds to experiences driven by volition [2,3].

Via the ESM, this study explored undergraduate students’ occupation characteristics, including mood, volition, activity satisfaction, mind-wandering, and social context. In this study, we added “momentary” before variables (e.g., momentary volition) which were immediately measured, via the ESM, in real life situations. With these momentary data, this study investigated how momentary personal causation, values, and interests contributed to momentary activity satisfaction and engagement in undergraduate students. A previous study reported that high volition measured by the Occupational Questionnaire was associated with high life satisfaction [4]. To closely observe the relationship between volition and satisfaction in the moment of performing an activity, the present study examined how momentary volition affected momentary activity satisfaction. Because volition influences how deeply people engage in activities [2,3,15], there is a possibility that low volition can lead to mind-wandering, indicating that attention is detached from the current activity [16]. This study investigated how momentary volition affected mind-wandering. We hypothesized that higher momentary volition would lead to higher activity satisfaction and less mind-wandering.

Moment-to-moment activity experiences comprise our lives, which implies that how we experience activities in the moment is linked to how we perceive our lives. With regard to life perspectives, life satisfaction and life balance would be related to momentary volition [4,17]. Life satisfaction is defined as “a global assessment of a person’s quality of life according to his chosen criteria” [18]. Previous studies have shown that life satisfaction is positively correlated with volition [4,19]. Life balance, another life perspective, is defined as “a satisfying pattern of daily activity that is healthful, meaningful, and sustainable to an individual within the context of his or her current life circumstances” [17]. The concept of life balance includes components of volition such as competence (personal causation), meaning (values), and enjoyment (interests) [17,20,21]. Based on previous reports on the relationship between volition elements, life satisfaction, and life balance, we investigated the association between momentary volition and life satisfaction as measured with the Satisfaction With Life Scale (SWLS) [22] and life balance measured by the Life Balance Inventory (LBI) [23]. We hypothesized that students who experienced high momentary volition would be more likely to show high levels of life satisfaction and life balance.

By investigating momentary volition, we could deepen our understanding of how clients experience activities that make up an individual’s narratives. With an understanding of moment-to-moment personal causation, values, and interests of undergraduate students, we can consider important volitional aspects that enhance their occupational participation and well-being. To provide implications of volition affecting occupational experience and life perspectives, the current study addressed the following two main research questions: (1) How do momentary volitional components (personal causation, values, and interests) contribute to momentary activity satisfaction and mind-wandering in undergraduate students? (2) Are volitional components, as measured moment-to-moment, associated with overall life satisfaction and life balance in undergraduate students?

To answer these questions, undergraduate students reported their momentary mood, activities, volition, activity satisfaction, mind-wandering, and social context via the ESM survey for one week. They then reported their overall life satisfaction measured with the SWLS and life balance measured with the LBI. At the event level of the ESM, the effect of volitional components on activity satisfaction and mind-wandering was analyzed. At the personal level, relationships of volition measured via the ESM with the SWLS and the LBI were analyzed.

## 2. Materials and Methods

### 2.1. Research Design

This study was part of a larger study of “the relationship between daily experience and well-being collected through smart phones,” which aimed to examine effects of momentary experience monitoring on participation and wellbeing. The present study was a cross-sectional observational study to investigate participants’ activities and perspectives in an ecologically valid way. Data were collected in September 2019. The project was approved by the Institutional Review Board of Soonchunhyang University. Informed consent was obtained from all participants.

### 2.2. Participants

A total of 45 Korean university students participated in this study. They were undergraduate students recruited via a convenience sampling method from a class of a university located in Chungcheongbuk-do Province, Korea. Participants who were over 18 years of age, had a smartphone, and completed the questionnaires were included. Three participants were excluded from the analysis due to missing data on the questionnaires. Valid data were collected from 42 participants. The sample size of this study was comparable to that of a previous study using the ESM [24]. All participants were compensated with a gift card (20,000 KRW ≈ 17 USD) for participating in this study.

### 2.3. Procedure

This study consisted of two phases. In the first phase, participants received the ESM survey about eight times a day for one week. On each ESM survey, participants reported their current mood, activity, activity type, personal causation, values, interests, activity satisfaction, mind-wandering, and social context. In the second phase, participants reported their life satisfaction and life balance by completing the SWLS and LBI on paper.

### 2.4. ESM

We used the ESM approach [13,14] to explore momentary experience while engaging in activities. In the ESM, participants are asked to answer on a series of questions (How is your mood now?, What are you doing now?, etc.) when they receive a notification which is usually sent randomly several times a day. The ESM is an ecologically valid approach because it collects real-time responses in real daily life. This study utilized the ESM to measure participants’ momentary experiences.

In this study, participants received a text message including the URL of the survey questions about eight times a day for one week. In each notification, participants were asked to answer on the ESM survey using a smartphone. The ESM survey was sent from 8 AM to 11 PM at random intervals of about two hours. A total of 55 messages was sent to each participant from 2:00 PM on 18 September to 2:00 PM on 25 September 2019. In the ESM survey via the URL in the text message, participants reported their momentary experiences. They were instructed to report their experiences at the time of notification as soon as possible.

### 2.5. Development of the ESM Survey

The ESM survey consisted of ten questions (Table 1) [4,16,23,25], including four questions from the previous ESM studies and six questions from questionnaires measuring clients’ occupations. These questions asked participants’ current mood (Q1), activity (Q2), activity type (Q3), volition including personal causation (Q4), values (Q5), interests (Q6), activity satisfaction (Q7), mind-wandering (Q8), social context asking whether they were interacting with someone (Q9) and if yes, who their interaction partner was (Q10). These items were translated and culturally adapted from English into Korean. All ESM questions were created on the SurveyMonkey platform.

The first question, “How is your mood now?” asked participants to rate their current feeling on a five-point scale (5, very good; 1, very bad). The question of mood was from the ESM item asking the level of happiness [16,26] which showed a relationship with events in daily life [27].

The second question of “What are you doing now?” asked participants to choose one of fifty-three activities from the LBI developed to encompass common everyday activities [23,25]. Some activity options were modified (from “eating nutritiously” to “eating,” from “getting adequate sleep” to “getting sleep,” and from “getting regular exercise” to “exercising”) so that they adequately referred to current activities. If participants could not find an appropriate answer among the 53 activities, they could select “other” to describe their current activity.

The following four questions asking about activity type and volition were from the Occupational Questionnaire [4]. Its original version can be found online (https://moho-irm.uic.edu/productDetails.aspx?aid=41 accessed on 27 August 2023). “I consider this activity to be:” asked participants to select the activity type, with options consisting of “work”, “daily living task”, “recreation”, and “rest”. Next, “I do this:” measured personal causation on a scale from “very well” (5) to “very poorly” (1). This question was shortened from the original question by removing the beginning phrase “I think” because participants would answer with their subjective thoughts. “For me, this activity is:” measured values on a scale from “extremely important” (5) to “total waste of time” (1). “How much do you like this activity?” measured interests on a scale from “like it very much” (5) to “strongly dislike it” (1). This question was modified to indicate “like” instead of “enjoy” to correspond to the word of option. On the original scale of questions asking volition in the Occupational Questionnaire, a positive answer was given a low point (e.g., 1 = very well, 5 = very poor). However, this study reversed the scale to assign high points to positive answers (e.g., 1 = very poor, 5 = very well), corresponding to other measurement scales for intuitive interpretation. The test–retest reliability of these questions in the Occupational Questionnaire was supported by good agreement ranging from 77% to 87% [4]. Validity was also established by comparing the Occupational Questionnaire with the diary, demonstrating an agreement of 84% to 86% [4].

Subsequently, “How much are you satisfied with this activity?” measured activity satisfaction on a scale from “very satisfied” (5) to “not at all satisfied” (1). Satisfaction of daily activities is one of the two main measurements of the Canadian Occupational Performance Measure [28], which assesses clients’ perceived performance and satisfaction with performance. Satisfaction with an activity is an important measurement aspect in Korean occupational therapy practice, which is reflected in the application frequency of the Canadian Occupational Performance Measure [29]. To measure mind-wandering, we used the question of “Are you thinking about something irrelevant to the activity that you are currently engaging in?” with an option of “yes” (1) or “no” (0). This question was from a previous study examining the relationship between mind-wandering and happiness [16].

The last two questions were about social context often identified in ESM studies [16,24,30]. “Are you interacting with someone right now?” asked participants to choose “yes” (1) or “no” (0) [16]. If “yes,” the following question, “With whom are you interacting right now?” asked participants to choose one of the options (friend, romantic partner, co-worker/classmate, senior at university, junior at university, professor, boss, sibling/relative, and parent) or “other” to describe an interaction partner [16,24].

### 2.6. Instruments

#### 2.6.1. Satisfaction with Life Scale

The SWLS measures an individual’s overall perception of life satisfaction [22]. The SWLS includes five items (e.g., “In most ways my life is close to my ideal”) rated on a 7-point Likert scale (7 = “strongly agree” to 1 = “strongly disagree”). The SWLS can be found at the website (http://labs.psychology.illinois.edu/~ediener/SWLS.html accessed on 27 August 2023). This study used the Korean-translated version of the SWLS [31]. For the Korean population, Cronbach’s alpha coefficient of the SWLS was 0.89, indicating a good internal reliability [32]. The convergent validity of SWLS in the Korean sample has also established, showing moderate correlation with the McGill Quality of Life questionnaire [33] (*r* = 0.58) [32].

#### 2.6.2. Life Balance Inventory

The LBI measures how an individual perceives their time in terms of daily activities [23,25]. The LBI presents 53 activities (e.g., “taking care of personal hygiene and bathing”, “doing things with friends”, and “shopping”) and asks the responder to check “yes” or “no” for whether he/she does or wants to do each activity. Next, for activities checked with “yes”, the responder compares the actual amount of time spent on each activity with the desired time. Options are “always less than I want” (1 point), “sometimes less than I want” (2 points), “about right for me” (3 points), “sometimes more than I want” (2 points), and “always more than I want” (1 point). The LBI can be found online (https://minerva.stkate.edu/LBI.nsf accessed on 20 August 2019). The original LBI asked for activities in the past month, which was modified to the past week in this study to match the ESM period. This study used the Korean version of the LBI [34], which showed suitable psychometric properties. For the Korean population, the split-half reliability of the LBI was 0.91, indicating excellent internal consistency [34]. The convergent validity of LBI in the Korean sample was also established, showing moderate correlations with the Perceived Stress Test [35] (*r* = 0.38) and SWLS (*r* = 0.34) [34].

### 2.7. Data Analysis

#### 2.7.1. Analysis at the Event Level

In the ESM, sampling data obtained within 15 min of notification were selected. Among responses to the “other” option in the activity question (Q2), homework was frequently described by participants. Thus, it was coded as a specific activity. Descriptive statistics were used to present summaries of the measurements. The mean and standard deviation or number and percentage were calculated for the overall ESM data. Descriptive statistics were then performed for each activity to understand participants’ volition for a specific activity.

To examine the effect of volition on activity satisfaction and mind-wandering in the moment, a hierarchical regression analysis was performed using ESM data. For activity satisfaction, the regression analysis included mood as a control variable in the first step. Personal causation, values, and interests were input in the second step. Likewise, on mind-wandering, we entered personal causation, values, and interests as independent variables after controlling for mood. By controlling for mood, genuine effects of volition on activity satisfaction and mind-wandering were analyzed.

#### 2.7.2. Analysis at the Personal Level

ESM data were summarized for each participant. Using these individual data, correlation analysis was performed to examine how momentary volition was related to life satisfaction and life balance measured by the SWLS and the LBI, respectively. 

MATLAB version 2021a (MathWorks, 2021) was used for sorting the data. All statistical analyses were conducted using SPSS version 25 (Armonk, NY, USA). The significance level was set at *p* < 0.05.

## 3. Results

### 3.1. Participants and Samplings

This study included 42 undergraduate students (13 males and 29 females; mean age = 21.24 years, *SD* = 1.41, range of 18 to 26 years). A total of 1641 sampling data points was collected. The mean number of responses was 39.07 (*SD* = 10.88, 71.0%). Data from 549 samplings were excluded because responses were not completed within 15 min of the notification. The final analysis included 1092 sampling data points. The average valid response per participant was 26.00 (*SD* = 8.68, 47.3%). The mean completion time was 3.83 min (*SD* = 3.72).

### 3.2. Descriptive Statistics

**Figure 1:**
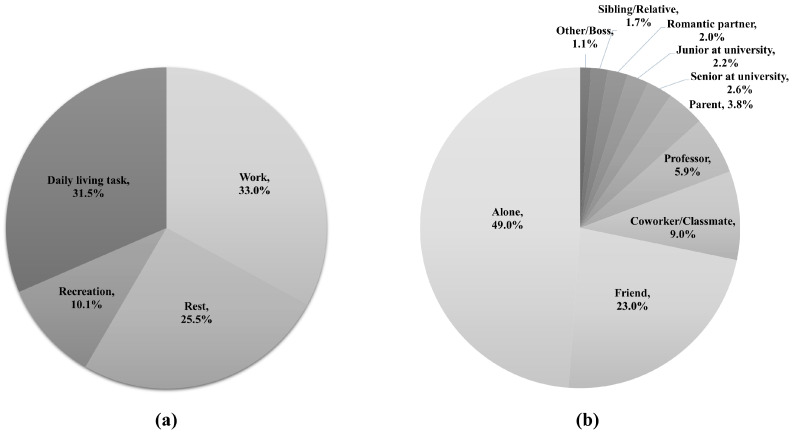
Activity type (**a**) and the person with whom the participant interacted (**b**).

Descriptive statistics of measurements are presented in Table 2 and Figure 1. Regarding the proportion of activity type, work (33.0%) was the most frequently reported activity type, followed by daily living task (31.5%, Figure 1a). Time alone (49.0%) was the most frequent response, followed by time with a friend (23.0%, Figure 1b). 

Table 3 illustrates results of the ESM including response count and volition in specific activities with ten and more responses. Relaxing was the most frequently reported activity (16.7%), followed by eating (12.0%) and taking the bus (12.0%). Watching TV, doing things with a spouse/significant other, and relaxing had the highest scores for personal causation, values, and interests, respectively.

### 3.3. Effects of Momentary Volition on Activity Satisfaction and Mind-Wandering

Hierarchical regression analysis was performed to investigate effects of momentary volition on momentary activity satisfaction and mind-wandering after controlling for the effect of mood. To examine the contribution of variables to activity satisfaction, the regression analysis included mood as a predictor variable in the first step. Personal causation, values, and interests were added in the second step. In the first step, involving mood, regression explained 28% of the variance (*F* [1, 1090] = 432.55, *p* < 0.001). Mood significantly predicted activity satisfaction (*β* = 0.53, *p* < 0.001). After adding personal causation, values, and interests in the second step, the model explained 61% of the variance, showing a variance change of 33% (*F* change [3, 1087] = 311.85, *p* < 0.001). All predictors, including mood (*β* = 0.18, *p* < 0.001), personal causation (*β* = 0.18, *p* < 0.001), values (*β* = 0.08, *p* < 0.001), and interests (*β* = 0.53, *p* < 0.001), were significant. Thus, volition made a unique contribution to activity satisfaction.

In another regression analysis on mind-wandering, mood was likewise entered in the first step and personal causation, values, and interests were added in the second step. In the first step involving mood, regression explained 2% of the variance (*F* [1, 1090] = 23.24, *p* < 0.001). Mood significantly predicted mind-wandering (*β* = −0.14, *p* < 0.001). In the second step adding personal causation, values, and interests, the model explained 5% of the variance, which changed the variance by 3% (*F* change [3, 1087] = 11.37, *p* < 0.001). Mood (*β* = −0.08, *p* = 0.03), personal causation (*β* = 0.08, *p* = 0.02), and interests (*β* = −0.22, *p* < 0.001) were significant predictors, but values were not significant (*β* = 0.03, *p* = 0.36). Mind-wandering was negatively affected by interests but positively correlated with personal causation.

### 3.4. Relationship of Momentary Volition with Life Satisfaction and Life Balance

This study analyzed correlation of momentary volition measured through the ESM with life perspective assessed by questionnaires on an individual level. Momentary interests were positively correlated with SWLS (*r* = 0.45, *p* = 0.003) and LBI scores (*r* = 0.31, *p* = 0.046). Personal causation showed a marginally significant positive correlation with LBI score (*r* = 0.28, *p* = 0.08). Other correlations were not significant (*rs* < 0.26, *ps* > 0.10). These results indicate that momentary interests were associated with the participants’ perceptions of how their lives were satisfied and balanced.

## 4. Discussion

This study demonstrated undergraduate students’ occupational characteristics using the ESM. Students’ momentary volition affected activity satisfaction and mind-wandering at that moment. In addition, momentary volition was related to overall life satisfaction and balance. Based on ecologically valid information, these findings provide evidence supporting that volition is linked to occupational experiences and life perspectives [2,3,36].

We found that personal causation, values, and interests all contributed to activity satisfaction, providing converging evidence for the relationship between volition and satisfaction [4]. A previous study reported overall volition and life satisfaction in elderly people, whereas the current findings showed an effect of volition on activity satisfaction of undergraduate students at each moment. Furthermore, this study demonstrated that volition uniquely contributed to satisfaction with daily activities by controlling for the effect of mood.

This study demonstrated that momentary volition was related to overall life perspective. High occupational interests were associated with high life satisfaction and balance, consistent with a previous finding showing that momentary pleasure was related to well-being [37]. Personal causation showed an only marginally significant correlation with life balance. Participants who perceived themselves as competent tended to live a more balanced life. This finding supports the life balance model, which describes that satisfying patterns of life consist of met needs for interests and competence [21]. The relationship between volition and life balance suggests that moment-to-moment experiences of occupational quality (interests and personal causation) are associated with subjectivity regarding occupational quantity (the amount of time). High volition may influence the perception that time was spent meaningfully, resulting in a good life balance. Alternatively, an appropriate allocation of time for daily activities could increase volition.

This study showed that each volition element had different relationships with occupational experiences and life perspectives. Interests were most consistently and strongly related to activity satisfaction and engagement, as well as overall life satisfaction and balance. Personal causation was a positive predictor of activity satisfaction. It could also lead to mind-wandering. Values showed significant relationships with activity satisfaction. Taken together, volition, especially interests, needs to be considered to improve undergraduate students’ occupations and life.

Current findings showed that interests were the most significant volition element, consistent with ESM research showing relationship between enjoyment of activity and quality of life. University students’ occupational enjoyment can affect their quality of life more strongly than other variables such as occupation type, location, social context, and health status [24]. Additionally, momentary enjoyment of activity can predict momentary quality of life in individuals with or without depression [38]. Momentary enjoyment of homework activity is positively correlated with self-esteem of adolescents [39]. Individuals who experience interests while engaging in an activity are more likely to choose the activity again because they feel rewarding emotions. A repeated activity choice provides opportunities to improve occupational skills [39,40,41]. Taken together, the current and previous studies suggest that interests are an influential volitional component in daily life.

A recent study has explored university students’ activities using the ESM [24] and revealed that students evaluate a moment positively when they participate in productive occupations, feel enjoyment during occupations, or are at home or with others. However, that study did not demonstrate individual differences in overall life perspectives according to momentary experiences. In the present study, by sorting ESM data by individual, we found that students who reported high momentary volition, especially interests, showed high life satisfaction and life balance.

The findings of this study showed how undergraduate students experienced what they were doing at the momentary level. The average of mood, personal causation, values, interests, and activity satisfaction were all above the midpoint of the scale, indicating that students reported positive rather than negative experiences. Participants reported mind-wandering in the moment 42.9% of the times sampled, which was similar to 46.9% reported in a previous study [26].

Mind-wandering could be explained by volition. Interests negatively predicted mind-wandering, meaning that when participants enjoyed an activity, they were less likely to think about things unrelated to the activity. This finding was consistent with previous findings that mind-wandering was related to an unhappy mind [16,26]. By focusing on volition rather than general states, this study showed that interests in an activity kept an individual’s mind on the activity. In contrast to interests, personal causation positively predicted mind-wandering. In other words, high competence in an activity was related to high mind-wandering during the activity. This result is plausible if there were more low-challenge activities than high-challenge ones. In low-challenge activities, people with high skills may experience boredom rather than concentration [14]. This speculation needs to be addressed in a further study that measures both skills and challenges [42,43].

Students categorized their activities into four types: work (33.0%), daily living task (31.5%), rest (25.5%), and recreation (10.1%). A previous study using the ESM found that Australian university students reported work most frequently at 32%, followed by quiet leisure (30%), self-care (13%), chores (9%), active leisure (8%), and sleep and rest (4%) [24]. Korean and Australian students similarly reported work most frequently. Rest and quiet leisure were the next frequent answers of Korean and Australian students, respectively. While comparison of results should be undertaken carefully due to differences in options between studies, Korean students seems to classify activities included in quiet leisure into rest rather than recreation. Time alone was reported the highest by Korean students (49.0%) and Australian students (46%). The second most frequent response was time with a friend in Korean students (23.0%) but time with a partner in Australian students (16%). This result could be attributed to a cross-cultural difference that Korean students felt closeness of romantic relationships less than students in the Western culture [44]. These results presented common or specific aspects of daily activities in university students across cultures.

This study has several limitations. First, because participants were recruited from a specific university, care should be taken when generalizing results to other populations. Second, the results might be underestimated due to a relatively small sample size. Therefore, a further study with more participants is needed to provide more solid findings. Third, a question on mind-wandering can reflect whether a person’s mind is in an activity or not, but not how deeply it is involved [45]. In a further study, a more precise measurement of the attention state could expand our understanding of the association between involvement and occupation. 

The current study provides implications for occupational therapy practitioners. The results offer further evidence that volition is related to occupational experiences and life perspectives. Among volition, interests were a strong element linked to the perception of occupation and life in undergraduate students. Interests have been suggested to motivate individuals to engage in an activity, which improve competence, especially in challenging activities [39,40,41]. These research findings imply that momentary interests need to be considered in occupational therapy interventions for satisfying occupational participation of undergraduate students.

## 5. Conclusions

This study demonstrated that momentary volition affected activity satisfaction and engagement and that it was related to life satisfaction and balance of undergraduate students. We measured momentary personal causation, values, and interests while performing occupations using the ESM. Momentary volition, especially interests, contributed to positive occupational experiences and life perspectives. This result is consistent with the Model of Human Occupation stating that personal causation, values, and interests are interwoven into the process of experience in doing things, which shapes our occupational narratives that is the subjective interpretation of lifetime. In this study of undergraduate students, momentary interests were relatively more emphasized when it came to perceiving their activity and life. Interests could be the focus of interventions to facilitate positive activity participation and subjective well-being. This study suggests that occupational therapy practitioners need to consider momentary interests to provide occupation-centered interventions for undergraduate students.

## Figures and Tables

**Table 1:** The experience sampling method survey.

Domain	Questions	Option
Mood	“How is your mood now?”	“very good” (5) to “very bad” (1)
Activity	“What are you doing now?”	fifty-three activities [25] and the “other” option
Activity type [4]	“I consider this activity to be:”	“work,” “daily living task,” “recreation,” or “rest”
Volition: Personal causation [4]	“I do this:”	“very well” (5) to ”very poorly” (1)
Volition: Values [4]	“For me, this activity is:”	“extremely important” (5) to “total waste of time” (1)
Volition: Interests [4]	“How much do you like this activity?”	“like it very much” (5) to “strongly dislike it” (1)
Activity satisfaction	“How much are you satisfied with this activity?”	“very satisfied” (5) to “not at all satisfied” (1)
Mind-wandering [16]	“Are you thinking about something irrelevant to the activity that you are currently engaging in?”	‘yes’ (1) or ‘no’ (0)
Social context [16]	“Are you interacting with someone right now?”	‘yes’ (1) or ‘no’ (0)
	“With whom are you interacting right now?”	friend, romantic partner, co-worker/classmate, senior at university, junior at university, professor, boss, sibling/relative, parent, and the “other” option.

**Table 2:** Descriptive statistics of measurements by the experience sampling method, the Satisfaction With Life Scale, and the Life Balance Inventory.

Measurements	Mean/*n*	*SD*/%
Experience sampling (*n* = 1092)		
Mood	3.29	0.98
Volition: Personal causation	3.79	0.87
Volition: Values	4.15	0.82
Volition: Interests	3.71	1.02
Activity satisfaction	3.43	1.13
Mind-wandering (*n*, %)	469	42.9%
Interaction (*n*, %)	559	51.2%
Questionnaires (*n* = 42)		
Satisfaction With Life Scale	18.45	6.34
Life Balance Inventory	2.26	0.35

**Table 3:** Descriptive statistics of response counts and volition in specific activities with ten and more responses from the experience sampling method.

Activity	Response Count		Volition					
			Personal Causation		Values		Interests	
	*n*	%	Mean	*SD*	Mean	*SD*	Mean	*SD*
Relaxing	182	16.7%	3.90	0.87	4.44	0.66	4.47	0.55
Eating	131	12.0%	4.19	0.83	4.47	0.61	4.40	0.59
Taking the bus	131	12.0%	3.80	0.85	3.68	1.15	2.70	1.01
Participating in educational opportunities	99	9.1%	3.23	0.73	3.92	0.68	3.10	0.58
Doing homework	68	6.2%	3.04	0.56	4.29	0.62	2.62	0.91
Doing things with friends	60	5.5%	3.92	0.81	4.17	0.69	4.12	0.76
Taking care of personal hygiene and bathing	42	3.8%	4.05	0.76	4.52	0.55	3.62	0.73
Watching TV	38	3.5%	4.32	0.70	3.92	0.78	4.32	0.57
Getting sleep	36	3.3%	4.19	0.95	4.58	0.50	4.39	0.60
Participating in groups	36	3.3%	3.58	0.91	3.86	1.02	3.44	0.91
Taking care of my appearance	34	3.1%	3.68	0.73	3.94	0.74	3.35	0.60
Working for pay	31	2.8%	4.00	0.58	4.32	0.65	3.00	1.06
Going to restaurants/bars	24	2.2%	4.04	0.62	4.13	0.80	4.17	0.70
Surfing the internet	19	1.7%	4.00	0.75	3.79	0.71	4.05	0.78
Playing games of skill	19	1.7%	4.05	0.62	3.79	0.86	4.42	0.61
Gaining competence in my job	17	1.6%	3.06	0.75	4.35	0.79	2.88	0.78
Doing things with family members	13	1.2%	4.31	0.85	4.46	0.66	4.46	0.52
Exercising	12	1.1%	3.67	0.89	3.92	0.79	3.83	0.83
Driving	11	1.0%	4.09	0.70	4.09	0.54	4.27	0.47
Doing things with spouse/ significant other	10	0.9%	4.00	1.15	4.60	0.52	4.40	1.26

## Data Availability

The data used to support the findings of this study are available from the corresponding author upon request.
